# Supplementary material for: Polysaccharides from Hericium erinaceus Fruiting Bodies: Structural Characterization, Immunomodulatory Activity and Mechanism
Source: Nutrients. 2022 Sep 9;14(18):3721. doi: 10.3390/nu14183721 (PMC9503163; doi:10.3390/nu14183721)
Supplement: Supplementary file 1 [file nutrients-14-03721-s001.zip › nutrients-1826272-supplementary.pdf]

Table S1. ^{1}H and ^{13}C NMR chemical shift data for HEP isolated from the fruiting bodies of *H. erinaceus*

Fractions	Residues		Proton or carbon					
			1	2	3	4	5	6
HEP-1	→6)-α-ᴅ-Glcp-(1→	H	5.01	3.64	3.74	3.58	3.78	3.96
		C	98.21	71.74	74.92	71.20	71.55	66.52
	→3)-β-ᴅ-Glcp-(1→↓	H	4.78	3.56	3.78	3.56	3.46	3.68,3.91
		C	103.31	73.33	-	68.83	75.92	60.77
	→3,6)-β-ᴅ-Glcp-(1→	H	4.76	3.57	3.78	3.56	3.71	4.26,3.86
		C	103.31	73.59	-	68.30	75.92	69.85
	→6)-α-ᴅ-Galp-(1→	H	5.04	3.91	4.08	3.93	4.26	3.76,3.96
		C	100.57	74.59	72.83	72.60	72.32	69.46
	→2,6)-α-ᴅ-Galp-(1→	H	5.04	3.78	4.04	3.71	4.17	3.64,3.95
		C	100.90	-	71.73	69.85	72.32	69.85
	→6)-β-ᴅ-Galp-(1→	H	4.93	3.46	3.58	3.86	3.62	3.61
		C	102.99	72.32	75.92	69.66	74.59	68.30
	α-ʟ-Fucp-(1→	H	5.15	3.74	3.86	4.04	4.14	1.20
		C	103.96	71.02	69.85	71.54	72.32	-
HEP-2	α-ᴅ-Glcp-(1→	H	5.51	4.08	3.88	3.84	3.65	3.76
		C	100.10	74.49	78.57	70.72	78.87	62.93
	→3)-β-ᴅ-Glcp-(1→	H	4.78	3.58	3.79	3.58	-	3.74,3.92
		C	103.27	73.50	85.96	69.39	75.99	61.38
	→3,6)-β-ᴅ-Glcp-(1→	H	4.78	3.58	3.79	3.58	3.71	4.20,3.82
		C	103.27	73.50	85.43	68.60	75.27	69.39
	→6)-α-ᴅ-Galp-(1→	H	5.01	3.91	4.05	3.92	4.20	3.75,3.98
		C	100.44	74.32	72.46	69.48	72.00	69.39
	→2,6)-α-ᴅ-Galp-(1→	H	5.05	3.79	4.05	3.71	4.16	3.64,3.92
		C	100.92	80.28	72.04	69.39	72.46	70.08
	1,2-Fruf	H	3.59	-	4.12	3.95-4.01	3.74	3.63
		C	60.00	103.11	76.84	74.05	80.27	62.43
HEP-3	α-ᴅ-Glcp-(1→	H	5.58	4.09	3.88	3.85	3.64	3.77
		C	100.13	74.40	78.60	70.73	78.86	63.01
	→3)-β-ᴅ-Glcp-(1→	H	4.78	3.57	3.77	3.53	3.51	3.69,3.92
		C	103.24	73.45	86.17	69.33	75.96	61.19
	→3,6)-β-ᴅ-Glcp-(1→	H	4.76	3.57	3.77	3.53	3.71	4.23,3.85
		C	103.24	73.45	86.17	69.33	75.96	70.03
	→6)-α-ᴅ-Galp-(1→	H	4.95	3.85	3.98	3.71	4.11	3.67,3.92
		C	100.23	74.40	72.49	69.33	72.06	69.09
	→2,6)-α-ᴅ-Galp-(1→	H	5.04	3.77	4.05	3.71	4.11	3.64,3.94
		C	100.23	79.98	72.15	69.33	72.49	70.03
	→6)-β-ᴅ-Galp-(1→	H	4.94	3.51	3.58	3.85	3.62	3.60
		C	102.95	72.49	75.96	69.33	75.01	68.55
HEP-4	α-ᴅ-Glcp-(1→	H	5.49	4.08	3.88	3.86	3.64	3.78
		C	100.22	74.45	78.51	70.70	78.87	62.97

	→6)-α-ᴅ-Glc-(1→	H	4.91	3.82	3.55	3.46	3.78	3.94
		C	97.55	72.84	74.15	71.17	70.21	61.72
	→3,6)-β-ᴅ-Glcp-(1→	H	4.76	3.55	3.79	3.55	3.72	4.26,3.88
		C	103.25	73.84	85.52	68.83	75.76	69.96
	→3)-β-ᴅ-Glcp-(1→	H	4.78	3.55	3.78	3.55	3.46	3.65,3.90
		C	103.25	73.84	86.15	68.25	75.76	60.95
HEP-5	α-ᴅ-Glcp-(1→	H	5.45	3.98	3.94	-	3.62	3.75
		C	100.1	74.4	78.5	70.7	78.8	62.9
	1,4-α-Glc	H	5.45	3.73	3.94	3.73	3.98	3.94
		C	101.13	72.42	72.77	78.15	74.58	61.81
	1,4,6-α-Glc	H	5.43	3.67	3.94	3.73	4.01	4.03
		C	100.85	72.78	73.06	78.98	74.23	68.56

- Not detected.

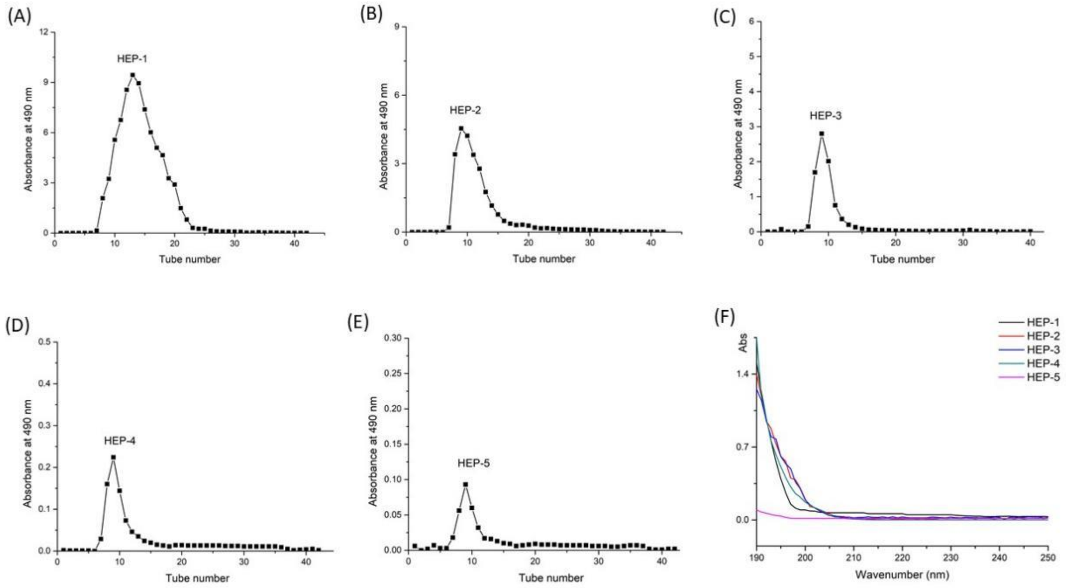

Figure S1. Elution curve of HEP fraction (A) HEP-1, (B) HEP-2, (C) HEP-3, (D) HEP-4 and (E) HEP-5 on Sephadex G-100 chromatography column and UV spectra of HEP-1, HEP-2, HEP-3, HEP-4 and HEP-5

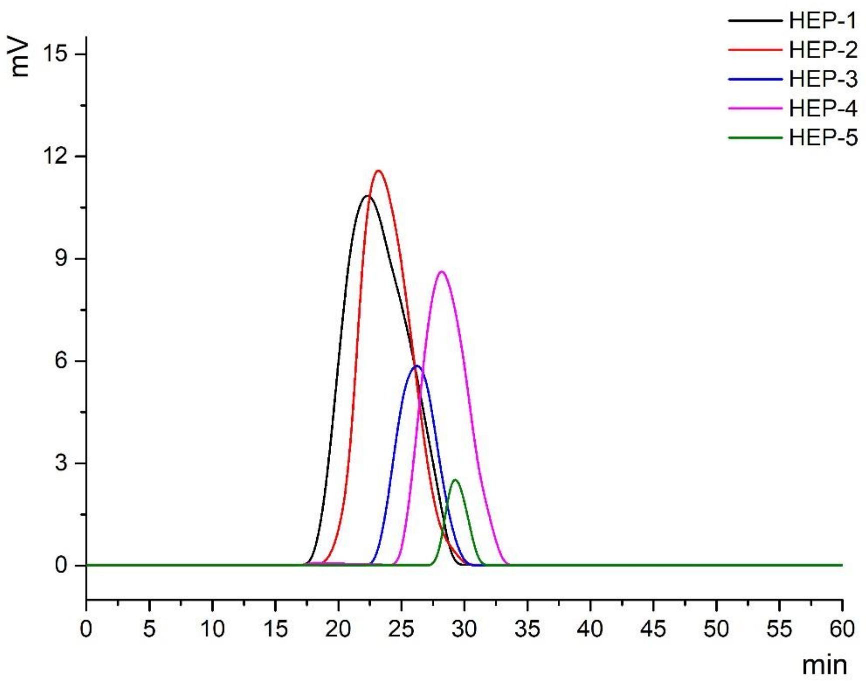

Figure S2. HPLC spectrum of relative molecular weights of HEP-1, HEP-2, HEP-3, HEP-4 and HEP-5

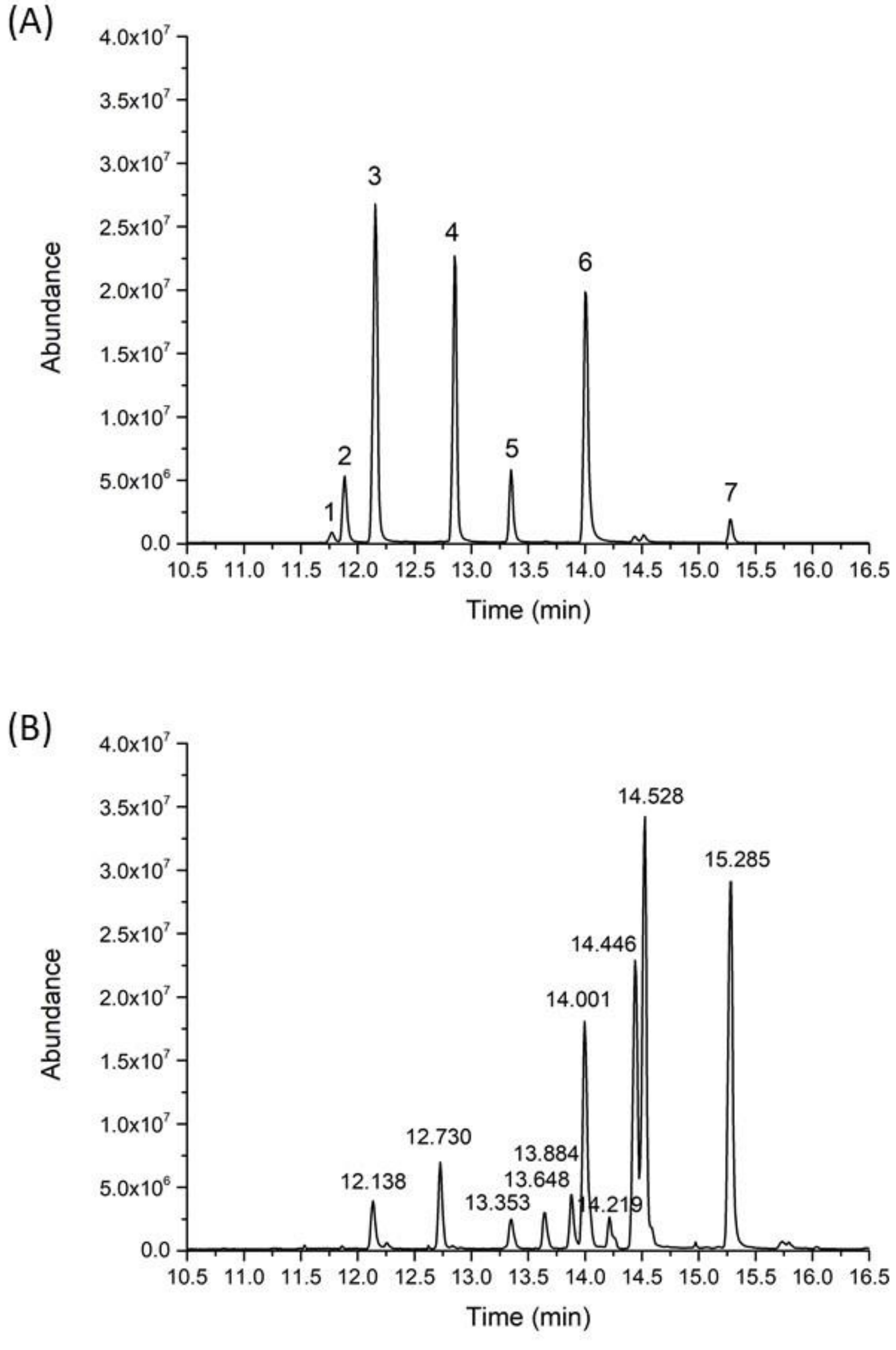

Figure S3. GC-MS spectrum of a standard solution of monosaccharides (A) and HEP (B). Peaks in (A) followed the order: (1) arabinose, (2) mannose, (3) fucose, (4) xylose, (5) rhamnose, (6) galactose, and (7) glucose
